# Navigating Primary Immune Thrombocytopenia During Pregnancy With Management Strategies and Considerations: A Comprehensive Review

**DOI:** 10.7759/cureus.67449

**Published:** 2024-08-21

**Authors:** S. M Dahiphale, Deepika Dewani, Manjusha Agrawal, Jayashree M Dahiphale, Garapati Jyotsna, Saloni, Rahul Desale

**Affiliations:** 1 Obstetrics and Gynaecology, Jawaharlal Nehru Medical College, Datta Meghe Institute of Higher Education and Research, Wardha, IND; 2 Neurology, Fortis Hospital, Mulund, Mumbai, IND; 3 Radiodiagnosis, Jawaharlal Nehru Medical College, Datta Meghe Institute of Higher Education and Research, Wardha, IND

**Keywords:** multidisciplinary care, autoimmune disorders, maternal-fetal health, thrombocytopenia management, pregnancy, primary immune thrombocytopenia (itp)

## Abstract

Primary immune thrombocytopenia (ITP) is an autoimmune disorder characterized by a reduction in platelet count due to autoantibody-mediated platelet destruction. ITP presents unique challenges during pregnancy, affecting both maternal and fetal health. This comprehensive review explores the pathophysiology, diagnosis, and management strategies of ITP in pregnant women, emphasizing the importance of individualized care. The incidence of ITP in pregnancy is significant, with potential complications including maternal hemorrhage and neonatal thrombocytopenia. Effective management is crucial to minimize these risks and ensure optimal outcomes. First-line treatments typically include corticosteroids and intravenous immunoglobulin (IVIG), with second-line options such as immunosuppressive agents and thrombopoietin receptor agonists. This review highlights the significance of multidisciplinary care and the need for careful monitoring and adjustment of treatment plans based on the severity of thrombocytopenia and the pregnancy stage. This review aims to enhance clinical decision-making and improve maternal and fetal outcomes in pregnancies complicated by ITP by providing a detailed analysis of current practices and emerging therapies.

## Introduction and background

Primary immune thrombocytopenia (ITP), formerly known as idiopathic thrombocytopenic purpura, is an autoimmune disorder characterized by a low platelet count (thrombocytopenia) due to the destruction of platelets by the immune system [1]. In ITP, autoantibodies target platelet surface antigens, leading to their destruction primarily in the spleen. The exact cause of autoantibody production is not well understood, but it is believed to involve a combination of genetic predisposition and environmental triggers [2]. ITP can manifest with a spectrum of symptoms, ranging from mild bruising and petechiae to severe bleeding episodes. The chronic form of ITP is more prevalent in adults, whereas the acute form often occurs in children following viral infections. It is relatively rare, with an estimated incidence of 1 to 6 per 100,000 people annually, and a prevalence of 10 to 20 per 100,000 individuals in the general population [1].

The incidence of ITP is approximately 1 to 2 per 1,000 pregnancies. The diagnosis and management of ITP in pregnant women present unique challenges due to the potential risks to both the mother and the fetus. Thrombocytopenia in pregnancy can arise from various causes, underscoring the importance of accurate diagnosis to initiate appropriate management strategies [3]. ITP during pregnancy poses significant risks for both the mother and the fetus. Maternal complications primarily revolve around the increased risk of bleeding, particularly during labor and delivery. Severe thrombocytopenia may necessitate platelet transfusions or immunosuppressive therapies to elevate platelet counts to safe levels [4]. The choice of treatment options during pregnancy requires careful consideration, balancing the need to manage maternal thrombocytopenia effectively while minimizing potential risks to the developing fetus [3].

For the fetus, maternal ITP can lead to complications such as neonatal thrombocytopenia. This condition arises when maternal antiplatelet antibodies cross the placenta and affect fetal platelets, potentially resulting in bleeding disorders in the neonate, including the serious complication of intracranial hemorrhage. The management of ITP during pregnancy, therefore, aims not only to protect the mother from bleeding risks but also to safeguard the fetus from the adverse effects of maternal autoimmunity [5]. The overarching purpose of this comprehensive review is to delve into the current understanding of primary ITP during pregnancy. By synthesizing the latest evidence and clinical insights, this review seeks to provide healthcare professionals with a detailed framework for effectively managing ITP in pregnant women. By addressing the complexities and challenges associated with this condition, the review aims to improve maternal health and fetal well-being outcomes.

## Review

Pathophysiology of ITP

**Figure 1:**
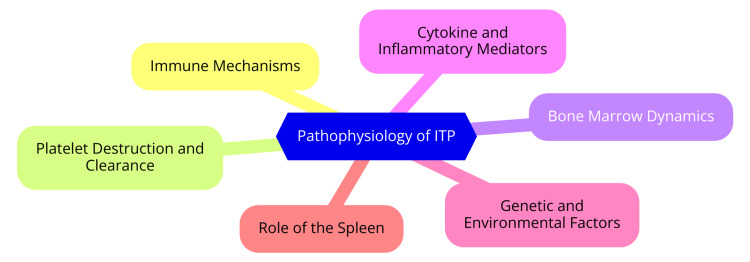
Pathophysiology of ITP Image credit: Dr. S. M. Dahiphale ITP: Immune thrombocytopenia

ITP is characterized primarily by a reduction in platelet count due to autoimmune mechanisms that lead to increased platelet destruction and impaired production. Understanding the pathophysiological mechanisms underlying ITP is crucial for effective management and treatment [6]. In ITP, the immune system generates autoantibodies, primarily immunoglobulin G (IgG), which target specific platelet membrane glycoproteins, such as the GPIIb/IIIa and GPIb/IX complexes [7]. These autoantibodies bind to platelets, marking them for destruction. This binding facilitates the phagocytosis of coated platelets by macrophages, predominantly in the spleen, where the autoantibodies are produced and destruction occurs. T cells and B cells play significant roles in the pathogenesis of ITP [7]. Activated B cells produce autoantibodies that opsonize platelets, while T helper cells, specifically the Th1 and Th17 subsets, contribute to the immune dysregulation observed in ITP. CD8+ cytotoxic T cells are also implicated in the direct destruction of platelets and megakaryocytes in the bone marrow, exacerbating thrombocytopenia. An imbalance between regulatory T cells (Tregs) and effector T cells results in a loss of immune tolerance, promoting the autoimmune response against platelets [8]. The primary mechanism of platelet destruction in ITP involves the recognition and clearance of autoantibody-coated platelets by macrophages through Fc receptor-mediated phagocytosis. This process is facilitated by the slow passage of platelets through the splenic sinusoids, where they encounter high concentrations of antibodies and Fc receptors on macrophages. Additionally, complement activation can lead to the direct lysis of platelets through the formation of the membrane attack complex (MAC), enhancing their destruction [9]. The destruction of platelets is often accompanied by impaired platelet production in the bone marrow. Although megakaryocytes (the precursor cells for platelets) may be present, their maturation and function can be compromised due to the autoimmune response. Autoantibodies can also target megakaryocytes, leading to their apoptosis and an inadequate increase in platelet production. The dysregulation of thrombopoietin (TPO), a key growth factor for megakaryocyte development, further contributes to the insufficient production of platelets in ITP [10]. The pathophysiology of ITP is illustrated in Figure 1.

Diagnosis of ITP in pregnancy

Clinical Presentation

ITP during pregnancy can present with a variety of signs and symptoms similar to those observed in non-pregnant individuals. Common symptoms include easy bruising, where individuals experience an increased tendency to bruise with minimal or no trauma. Patients may also present with petechiae, small red or purple spots on the skin caused by bleeding. Additionally, prolonged bleeding from minor cuts or procedures, as well as mucosal bleeding, such as nosebleeds or bleeding gums, can occur. Interestingly, some women may be asymptomatic and discover their condition only through routine blood tests, underscoring the importance of regular monitoring during pregnancy [11]. When diagnosing ITP, it is crucial to consider a differential diagnosis for thrombocytopenia in pregnant women. The most common alternative diagnosis is gestational thrombocytopenia, typically presenting with mild platelet reductions and not associated with adverse outcomes for the mother or fetus. Other conditions to consider include preeclampsia and HELLP (Hemolysis, Elevated Liver enzymes, and Low Platelet count) syndrome, characterized by hypertension and additional symptoms. Thrombotic thrombocytopenic purpura (TTP) should also be ruled out, as it presents with microangiopathic hemolytic anemia and organ dysfunction. Additionally, aplastic anemia and various bone marrow disorders, such as leukemia or myelodysplastic syndromes, can lead to similar laboratory findings. Accurate diagnosis is crucial, as these conditions can significantly impact maternal and fetal health [12].

Diagnostic Criteria and Tools

Diagnosing ITP during pregnancy primarily relies on clinical evaluation and laboratory testing. A complete blood count (CBC) is crucial for assessing platelet levels, with a count below 100,000/mm³ raising suspicion for ITP, particularly if there is a declining trend. In some cases, testing for specific antiplatelet antibodies can help confirm the diagnosis; however, the results may often be inconclusive during pregnancy due to elevated levels of platelet-associated IgG in many women [3]. While a bone marrow examination is not routinely performed for all suspected cases of ITP, it may be indicated if the diagnosis remains uncertain or if there is suspicion of a bone marrow disorder. This examination helps rule out conditions such as leukemia or aplastic anemia, which can also present with thrombocytopenia [13]. The diagnosis of ITP is fundamentally one of exclusion, requiring healthcare providers to rule out secondary causes of thrombocytopenia. Potential secondary causes include infections like HIV, hepatitis, and *Helicobacter pylori*, as well as autoimmune diseases such as lupus, which can also result in low platelet counts. Certain medications may also induce thrombocytopenia. A thorough patient history, including previous platelet counts and a history of bleeding disorders, is critical for making an accurate diagnosis. In cases with no prior history, significant thrombocytopenia in early pregnancy, particularly with a declining trend, is more indicative of ITP [14].

Management strategies for ITP in pregnancy

Managing ITP during pregnancy involves carefully balancing treatment options to ensure the safety of both the mother and the fetus, tailored to the severity of the condition. First-line treatments typically include corticosteroids and intravenous immunoglobulin (IVIG). Corticosteroids, such as prednisone, are the primary treatment choice for pregnant women with ITP [15]. The initial dosage usually ranges from 10 to 20 mg per day, with a response typically beginning within 2 to 14 days and peaking between 4 and 28 days. IVIG is another first-line option, particularly useful when a rapid increase in platelet counts is necessary, such as in preparation for delivery. This treatment can produce an initial response within one to three days, with peak effects occurring in two to seven days [16]. If first-line treatments are ineffective or insufficient, second-line treatments may be considered. Immunosuppressive agents, such as azathioprine, can be used; however, their safety during pregnancy remains a topic of debate due to potential risks to the fetus. Anti-D immunoglobulin is another option, especially for Rh-positive mothers with anti-D antibodies, but its use is limited and requires careful case assessment. Splenectomy, while generally reserved for women with severe, refractory ITP, may be considered during the second trimester when risks to both mother and fetus can be better managed [17]. TPO receptor agonists (TPO-RAs), such as eltrombopag and romiplostim, have shown promise in treating ITP, but their safety profile during pregnancy is not well established. Consequently, these agents are not routinely recommended for use in pregnant women. Given the limited data on their effects during pregnancy, caution is advised when considering TPO-RAs as a treatment option [18]. Treatments such as platelet transfusions and plasmapheresis may be necessary in emergencies. Platelet transfusions can be critical in acute settings where rapid correction of platelet counts is required, such as before surgical procedures or in cases of severe bleeding. Plasmapheresis, which involves the removal of circulating antibodies, can also be utilized in severe cases of ITP, although it is less commonly employed during pregnancy [19]. The management of ITP during pregnancy requires a multidisciplinary approach, with careful monitoring and individualized treatment plans to ensure maternal and fetal safety. Regular follow-up and assessment of platelet counts are crucial in guiding therapy and making timely decisions regarding delivery and anesthesia options. By employing a comprehensive strategy, most women with ITP can achieve successful pregnancies while minimizing risks [20].

Special considerations in pregnancy

Monitoring and Follow-Up

Regular monitoring of platelet counts is crucial for managing ITP during pregnancy. The recommended monitoring schedule typically includes checking platelet levels every four weeks during the first and second trimesters, every two weeks after 28 weeks, and weekly during the final month of pregnancy [5]. This structured approach enables healthcare providers to closely track platelet count changes and make timely treatment adjustments as necessary. Treatment plans should be individualized based on the patient's platelet levels to maintain a safe count for both the mother and the fetus. Close collaboration between obstetricians and hematologists is essential to ensure effective management and promptly address emerging concerns [21].

Maternal and Fetal Risks

Thrombocytopenia associated with ITP can pose significant risks for both the mother and the fetus. For mothers, the primary concern is an increased risk of bleeding, particularly during delivery. Severe thrombocytopenia, defined as a platelet count below 20,000/µL, can lead to life-threatening hemorrhage, necessitating careful monitoring and proactive management [3]. For the fetus, there is a risk of fetal/neonatal alloimmune thrombocytopenia (FNAIT), a condition in which maternal antibodies cross the placenta and target fetal platelets. This can result in severe thrombocytopenia and potentially life-threatening complications, such as intracranial hemorrhage in the neonate. Therefore, regular monitoring and appropriate treatment are essential to minimize these risks and ensure the safety of both mother and child [22].

Labor and Delivery Planning

Planning for labor and delivery in the context of ITP requires careful consideration of the mother's platelet levels and overall health. The mode of delivery should be determined based on obstetric indications, with specific platelet count thresholds guiding decisions. Vaginal delivery is generally recommended if platelet counts are 30,000/µL or higher, while cesarean delivery may be preferred if platelet counts are 50,000/µL or above [3]. Anesthetic considerations are also critical; neuraxial anesthesia, such as epidurals, is typically considered safe if platelet counts are between 70,000 and 80,000/µL, although there is some debate regarding this threshold. Peripartum management of thrombocytopenia may involve administering corticosteroids, IVIG, or platelet transfusions to maintain safe platelet levels during delivery. Effective communication among obstetricians, hematologists, and anesthesiologists is essential to ensure a coordinated approach prioritizes the mother’s and baby’s health and safety [23].

Case studies and clinical outcomes

Several case studies have demonstrated successful management strategies for primary ITP during pregnancy, highlighting the importance of a multidisciplinary approach. One notable case involved a patient with severe ITP who was treated with corticosteroids and IVIG. This regimen enabled her to achieve a safe vaginal delivery at 35 weeks, resulting in good maternal and neonatal outcomes. This case underscored the necessity of close monitoring and collaboration among obstetricians, hematologists, and anesthetists throughout the pregnancy [24]. Another compelling case featured a 29-year-old primigravida diagnosed with ITP during pregnancy. Effective communication and teamwork between her obstetric and hematology teams facilitated a smooth antenatal course. Ultimately, she underwent a cesarean delivery at 35 weeks due to increasing resistance to treatment, but both she and her baby fared well. Additionally, a case involving a 17-year-old primigravida with severe gestational thrombocytopenia demonstrated the critical need for urgent management. With limited time before delivery, the patient was successfully treated with corticosteroids and IVIG, emphasizing the importance of timely intervention in high-risk scenarios [25]. Despite generally positive outcomes associated with ITP in pregnancy, complications can arise if the condition is not effectively managed. Maternal hemorrhage, fetal thrombocytopenia, and fetal intracranial hemorrhage are serious risks associated with severe ITP. Furthermore, neonates born to mothers with ITP may experience transient thrombocytopenia, with a small risk (less than 1%) of intracranial hemorrhage. The use of corticosteroids during the first trimester has been linked to a slight risk of fetal orofacial cleft palate, while administration in the third trimester may lead to maternal complications such as diabetes, weight gain, and hypertension [26]. These cases emphasize the importance of maintaining safe platelet counts, especially as delivery approaches, and avoiding unnecessary interventions like cesarean sections based solely on platelet levels. The key takeaway is that careful planning, close follow-up, and individualized treatment strategies are essential to minimize risks and enhance outcomes for both mother and child [27]. Overall, outcomes for pregnancies in women with ITP are encouraging. Most pregnancies result in successful outcomes for both mothers and infants, with many neonates born to mothers with gestational thrombocytopenia showing excellent health and rarely developing significant complications. The long-term prognosis for mothers with ITP is generally favorable, with many experiencing remissions over time. This positive outlook highlights the effectiveness of modern management strategies and the importance of a collaborative approach to care [28]. While ITP in pregnancy poses unique challenges, most women can achieve successful pregnancies with appropriate management. Close monitoring, individualized treatment plans, and a multidisciplinary approach are crucial for optimizing maternal and neonatal outcomes [29].

Future directions and research

Innovative Treatments and Therapies

Recent advancements in managing ITP have introduced several innovative treatments that may significantly transform patient care. Among these emerging therapies, avatrombopag, an oral TPO-RA, stands out for its potential. This drug has shown efficacy comparable to existing treatments, offering advantages such as fewer food interactions and reduced monitoring requirements. Its oral administration could improve patient adherence and convenience, making it a valuable addition to the ITP treatment options [30]. Another significant development is fostamatinib, a spleen tyrosine kinase inhibitor targeting the mechanisms underlying platelet destruction. Currently under investigation, this agent may be particularly beneficial for patients who have not responded to conventional therapies. Additionally, anti-CD38 therapy, exemplified by daratumumab, is being explored for its potential to deplete long-lived plasma cells responsible for producing antiplatelet autoantibodies. This novel approach could offer a new method for managing ITP by directly addressing the autoimmune aspect of the disease [31]. Moreover, researchers are examining Bruton tyrosine kinase inhibitors, which may modulate immune responses and provide a new therapeutic option for ITP patients. The potential of gene editing technologies, such as clustered regularly interspaced palindromic repeats (CRISPR)-Cas9, is also being explored to correct genetic mutations associated with ITP. Developing bispecific antibodies targeting both autoreactive immune cells and platelets also represents an exciting frontier in ITP therapy. These innovative treatments promise to improve patient outcomes and expand the therapeutic options available for managing this complex condition [32].

Potential for Personalized Medicine in ITP Management

The future of ITP management is increasingly moving towards personalized medicine, which tailors treatment strategies to the unique characteristics of each patient. Genetic testing is expected to be crucial in this shift by identifying specific mutations that affect disease severity and treatment response. Understanding patients' genetic and immunological profiles will enable healthcare providers to develop more effective and customized treatment plans, optimizing therapeutic outcomes while minimizing adverse effects [33]. Additionally, integrating advanced biomarker research will likely enhance the ability to predict disease progression and treatment efficacy. Identifying biomarkers associated with treatment response will allow clinicians to make more informed decisions regarding therapy selection, leading to improved management strategies. Personalized medicine has the potential to enhance the effectiveness of existing treatments; it paves the way for developing novel therapies tailored to specific patient subsets with distinct pathophysiological features [34].

Areas for Further Research

Despite the promising advancements in ITP treatment, substantial gaps remain in our understanding of the disease's pathophysiology. Comprehensive research is essential to elucidate the roles of various immune cell types, genetic factors, and environmental influences in the onset and progression of ITP. Such insights are critical for developing targeted therapies that address the underlying causes of the disease, rather than merely managing its symptoms [35]. To address these knowledge gaps, large-scale studies and clinical trials are imperative. Although numerous ongoing trials investigate new therapies and treatment combinations, additional, comprehensive studies are needed to establish long-term efficacy and safety profiles. Collaborative efforts among researchers, clinicians, and patients are vital for developing innovative treatment strategies and improving the overall management of ITP. By fostering a robust research environment, the medical community can enhance the understanding of ITP and ultimately improve outcomes for patients facing this challenging condition [36].

## Conclusions

In conclusion, navigating primary ITP during pregnancy requires a nuanced and multidisciplinary approach to ensure the safety and well-being of both mother and fetus. The management of ITP in pregnant women presents unique challenges due to the dual need to control maternal thrombocytopenia and mitigate potential risks to the fetus. Effective management involves accurate diagnosis, careful monitoring, and individualized treatment strategies that balance the benefits and risks of various therapeutic options. The potential complications associated with ITP, such as increased maternal bleeding risk and neonatal thrombocytopenia, underscore the importance of a thorough understanding of the condition and its implications. This comprehensive review aims to equip healthcare professionals with the necessary knowledge and tools to manage ITP in pregnancy effectively, thereby optimizing outcomes for both mother and child. Continued research and advancements in therapeutic approaches are essential to further refine management strategies and improve the quality of care for pregnant women with ITP.
